# PRMT5 prognostic value in cancer

**DOI:** 10.18632/oncotarget.26883

**Published:** 2019-05-07

**Authors:** Hanine Lattouf, Coralie Poulard, Muriel Le Romancer

**Affiliations:** ^1^ INSERM U1052, Centre de Recherche en Cancérologie de Lyon, Lyon, France; ^2^ CNRS UMR5286, Centre de Recherche en Cancérologie de Lyon, Lyon, France; ^3^ Université Lyon 1, Lyon, France; ^4^ Lebanese University, EDST (Molecular Tumor-genesis and Anticancer Pharmacology), Hadath, Lebanon

**Keywords:** arginine methylation, PRMT5, breast cancer, biomarker

## Abstract

Protein arginine methyltransferases (PRMTs) catalyze the methylation of arginine residues on both histones and non-histone proteins. PRMT5, a member of the PRMT family, is overexpressed in a wide variety of cancers and its activity is associated with cell transformation. Moreover, its expression is associated with a decrease in patient survival in several cancers, a rationale for developing highly potent inhibitors of its enzymatic activity. However, most studies do not take into account the subcellular localization of PRMT5, which can modify its properties. Indeed, our team recently showed that PRMT5 nuclear expression is associated with prolonged survival. These results corroborated findings in prostate cancer, in which the nuclear fraction of PRMT5 was responsible for inhibiting cell growth, while the cytoplasmic fraction promoted cell growth. In conclusion, this criterion should be evaluated prior to administering PRMT5 inhibitors, which may have adverse effects.

Protein arginine methyltransferases (PRMTs) catalyze the methylation of arginine residues on a variety of proteins including histones and non-histone proteins. Arginine methylation is a frequent post-translational modification involved in various cellular processes, such as DNA transcription, mRNA splicing, signal transduction, protein interaction and subcellular protein localization [1, 2]. Members of the family are classified into three functional groups depending on the type of methylation they catalyze. Type 1 PRMTs catalyze monomethylation and asymmetrical dimethylation of arginine residues, while type II PRMTs induce monomethylation and symmetrical demethylation. In contrast, type III PRMTs trigger only monomethylation [2]. PRMT5, the major type II PRMT is essential for normal development [3] and was shown to be overexpressed in a wide variety of cancers, such as prostate, breast, lung and colon cancer [1].

In this context, most studies have highlighted that PRMT5 overexpression is associated with a decrease in patient survival and an increase in ovarian, lung, multiple myeloma and breast tumor growth [4-7] (Figure 1A, 1B). Furthermore, an increase in its activity is associated with cell transformation, exposing PRMT5 as a suitable druggable target for treating cancer [3]. Consequently, highly potent and selective PRMT5 inhibitors, such as EPZ015666, were developed, displaying anti-tumor properties in animal models [8], and have recently entered clinical trial.

**Figure 1:**
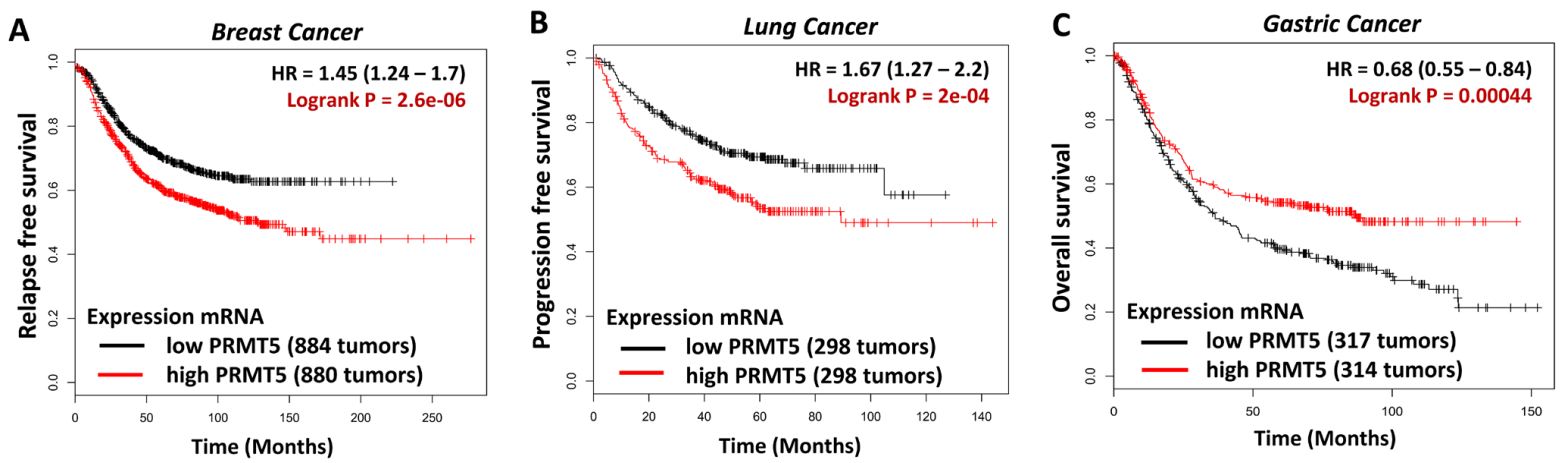
Kaplan-Meier analysis comparing survival of a cohort of breast (A), lung (B) or gastric cancer (C), separated into low or high PRMT5 expression as indicated using appropriate databases [13].

However, we would like to raise awareness of the dangers of indiscriminately using such inhibitors for different types of cancers. Indeed, database analysis highlighted that the expression of PRMT5 can also be associated with prolonged survival for instance in gastric cancers (Figure 1C), at the opposite of the breast and lung cancers (Figure 1A, 1B). Moreover, findings derived from biomarker mRNA expression levels do not take in account the subcellular localization of proteins, which can modify their properties. This has been demonstrated for the protein encoded by the tumor suppressor gene *LKB1*, since its nuclear expression has been associated with increased survival in breast cancer, whereas its cytoplasmic expression is associated with a decrease in survival [9]. More recently, similar results were obtained for the transcriptional coregulator RIP140 [10], and by our own group for PRMT5, the nuclear expression of which is of good prognosis and associated with prolonged survival. Furthermore, our study demonstrated a positive correlation between nuclear PRMT5 and LKB1, illustrated by an increase in survival exclusively in LKB1-positive patients displaying a nuclear PRMT5 expression [11]. These findings, suggest a functional relationship between these proteins which merits further investigation. Finally, our results also corroborated findings in prostate cancer, in which the nuclear fraction of PRMT5 was detected in benign prostate epithelial cells and was responsible for inhibiting cell growth and limiting their proliferation, while the cytoplasmic fraction that was found in cancerous prostate tissues and promoted cell growth [12]. 

Overall these observations emphasize the importance of PRMT5 localization in regulating its activity and its oncogenic properties. This criterion should be evaluated prior to administration of PRMT5 inhibitors, which may thus have adverse effects. It would be of interest to understand the mechanisms involved in PRMT5 regulation in the nucleus of tumoral cells and to investigate whether its dual prognostic value can be extended to other cancers.
